# Dietary Stearic Acid Leads to a Reduction of Visceral Adipose Tissue in Athymic Nude Mice

**DOI:** 10.1371/journal.pone.0104083

**Published:** 2014-09-15

**Authors:** Ming-Che Shen, Xiangmin Zhao, Gene P. Siegal, Renee Desmond, Robert W. Hardy

**Affiliations:** 1 Department of Pathology, University of Alabama at Birmingham, Birmingham, Alabama, United States of America; 2 Departments of Cell, Developmental & Integrative Biology and Surgery, University of Alabama at Birmingham, Birmingham, Alabama, United States of America; 3 Department of Medicine, Division of Preventive Medicine, University of Alabama at Birmingham, Birmingham, Alabama, United States of America; University of the Witwatersrand, South Africa

## Abstract

Stearic acid (C18:0) is a long chain dietary saturated fatty acid that has been shown to reduce metastatic tumor burden. Based on preliminary observations and the growing evidence that visceral fat is related to metastasis and decreased survival, we hypothesized that dietary stearic acid may reduce visceral fat. Athymic nude mice, which are used in models of human breast cancer metastasis, were fed a stearic acid, linoleic acid (safflower oil), or oleic acid (corn oil) enriched diet or a low fat diet ad libitum. Total body weight did not differ significantly between dietary groups over the course of the experiment. However visceral fat was reduced by ∼70% in the stearic acid fed group compared to other diets. In contrast total body fat was only slightly reduced in the stearic acid diet fed mice when measured by dual-energy x-ray absorptiometry and quantitative magnetic resonance. Lean body mass was increased in the stearic acid fed group compared to all other groups by dual-energy x-ray absorptiometry. Dietary stearic acid significantly reduced serum glucose compared to all other diets and increased monocyte chemotactic protein-1 (MCP-1) compared to the low fat control. The low fat control diet had increased serum leptin compared to all other diets. To investigate possible mechanisms whereby stearic acid reduced visceral fat we used 3T3L1 fibroblasts/preadipocytes. Stearic acid had no direct effects on the process of differentiation or on the viability of mature adipocytes. However, unlike oleic acid and linoleic acid, stearic acid caused increased apoptosis (programmed cell death) and cytotoxicity in preadipocytes. The apoptosis was, at least in part, due to increased caspase-3 activity and was associated with decreased cellular inhibitor of apoptosis protein-2 (cIAP2) and increased Bax gene expression. In conclusion, dietary stearic acid leads to dramatically reduced visceral fat likely by causing the apoptosis of preadipocytes.

## Introduction

Numerous studies have identified obesity as a risk factor for postmenopausal breast cancer [1], [2]. Obesity is also associated with both an increased tumor burden [3] and higher grade tumors [4]. Importantly, in premenopausal and postmenopausal breast cancer, women's obesity is associated with poorer outcomes and/or increased mortality [1], [2], [4]–[7] although the effect of body mass index (BMI) on mortality may be less in younger women compared to postmenopausal women [8]. Based on 4 studies [2], [5]–[7] the risk of patients with breast cancer dying is increased between 40–70% by obesity and these increases are highly significant. A growing body of literature have found that visceral adipose tissue (VAT) is also related to cancer survival [9]–[11]. This may be due to differences between VAT and other fat depots with respect to their cytokine profile which favors lipolysis, inflammation, angiogenesis, and insulin resistance [12]. In a recent study we found that dietary stearic acid reduced metastasis tumor burden in an athymic (nude) mouse breast cancer metastasis model [13]. Stearic acid, an 18-carbon long chain saturated fatty acid (SFA), is found in significant concentrations in several foods in the western diet including beef, chocolate, and milk fats. Although we have investigated potential mechanisms whereby dietary stearic acid may inhibit metastasis, they have not been confirmed *in vivo*. A serendipitous observation in these studies suggested that perhaps dietary stearic acid reduced VAT. The present studies were designed to confirm and expand this observation and to begin to understand the mechanism driving these changes.

## Materials and Methods

All *in vivo* animal procedures were approved by the Institutional Animal Care and Use Committee (IACUC), University of Alabama at Birmingham (UAB).

### Reagents

Stearic acid (≥98.5%), oleic acid (≥99%), linoleic acid (≥99%), diatomaceous earth, insulin, dexamethasone, 3-isobutyl-1-methyl-xanthine, and fatty acid free bovine serum albumin (BSA) were obtained from the Sigma-Aldrich Chemical Co. (St. Louis, MO). Trypan blue was purchased from Eastman Kodak Company (Rochester, NY). Oil Red O was acquired from Rowley Biochemical (Rowley, MA) and Hematoxylin I was obtained from Richard-Allan Scientific (Kalamazoo, MI).

### Animals and Diets

Since our previous studies used athymic nude mice and these mice are commonly used for xenograft experiments using human cancer cells, we used these same mice to confirm our hypothesis. Three-to-four week old female athymic mice were purchased from Harlan Sprague Dawley, Inc. (Indianapolis, IN) and were maintained in microisolater cages in pathogen-free facilities. Animals were divided randomly into four groups of 10 mice each, and were placed on one of four diets: a low fat diet (5% corn oil diet) comparable to normal rodent chow, a 20% safflower oil diet, a 17% corn oil/3% safflower oil diet and a 17% stearic acid/3% safflower oil diet. The stearic acid-rich diet used in these studies contained a minimum amount of essential fatty acids required for normal growth and development, and dietary stearic acid as the primary fatty acid. This diet minimizes the confounding effects of other fatty acids while not affecting total body weight [13], [14]. These diets were prepared by Harlan-Teklad (Madison, WI) and details have been published [13].

The animals were fed *ad libitum* for 18 weeks and 3 days, and the amount of food consumed was recorded. Mice were anesthetized with 3% isoflurane in 2.5% O_2_ and weighed weekly. At 18 weeks and 3 days the mice were sacrificed and the brain, heart, lungs, kidneys, liver, and abdominal fat were collected.

### Dual energy X-ray absorptiometry (DXA)

Mice were scanned using the GE Lunar PIXImus dual-energy X-ray absorptiometer (Fitchburg, WI) utilizing software version 1.45 after 18 weeks on their respective diets (1 day for DXA). Using an IACUC approved procedure, each animal was placed in an airtight container and anesthetized using the microdrop method with Isoflurane (4%). Once the mouse was immobile and breathing steadily, it was placed in a prostrate position on the DXA imaging plate and scanned. During the scan the mouse remained anesthetized using an Isoflurane (3%) and oxygen (500 ml/min) mixture. Each scan took less than 5 minutes. Data obtained from these scans included bone mineral content (BMC), bone mineral density (BMD), lean mass and fat mass.

### Quantitative magnetic resonance (QMR)

*In vivo* body composition (total body fat and lean tissue) of mice was also determined the day following DXA using an EchoMRI 3-in-1 quantitative magnetic resonance (QMR) composition analyzer (Echo Medical Systems, Houston, TX). Each animal was placed in a clear tube that restricted vertical movement, but allowed constant airflow. No anesthesia was required. The tube was inserted into the instrument and scanning was initiated.

### Measurement of serum glucose, insulin, leptin, monocyte chemotactic protein-1 (MCP-1), interleukin-6 (IL-6), and adiponectin

Due to the limited amount of serum available from the mice, we selected 6 analytes that would address the two most likely mechanisms (insulin resistance and increased inflammatory cytokines). We analyzed IL-6 and MCP-1 (markers generally associated with inflammation) in mouse serum using Meso Scale Discovery (Gaithersburg, MD) mouse cytokine assay ultra-sensitive kits. The coefficient of variation (CV) for these assays was 9% and 3%, respectively. Mouse serum leptin (associated with obesity, appetite and angiogenesis), insulin and adiponectin (associated with improved insulin sensitivity) were measured using Millipore (Billerica, MA) radioimmunoassay kits with CVs of 7%, 4% and 2% respectively. Serum glucose was measured by a glucose oxidase assay run on a Stanbio Sirrus instrument (Stanbio Laboratory, Boerne, TX). This assay had a 3% CV.

### Paraffin section and H&E staining

Paraffin sections were prepared as described previously [15]. Briefly, 10% filtered and buffered formalin fixed samples (abdominal fat, kidney and liver) were processed with a VIP 1000 tissue processor (Sakura-Finetek, Torrance, CA) through graded alcohols and xylene, then embedded into paraffin blocks. Five micron sections were cut on a Leica 2135 rotary microtome (Leica Microsystems, Bannockburn, IL), air-dried, deparaffinized and stained with Hematoxylin & Eosin stains (Richard Allen Scientific, Kalamazoo, MI).

### 3T3L1 Cell Culture

3T3L1 mouse preadipocyte, fibroblast cells (American Type Culture Collection (ATCC), CL-173) were maintained according to the ATCC recommended protocol, in Dulbecco's Modified Eagle's Medium (DMEM) containing 10% fetal bovine serum and antibiotics (M1 medium). Differentiation to adipocytes was performed according to standard procedures [29]. Briefly, 3T3L1 fibroblasts were seeded at 30% confluence and grown to >90% confluence. After reaching >90% confluence, the M1 medium was replaced with M1 medium contained insulin (5 µg/ml), dexamethasone (0.25 µM), and 3- isobutyl-1-methyl-xanthine (0.5 mM). Two days later, the cells were changed to M1 medium with insulin (5 µg/ml) for another 2 days. Cells were then maintained in M1 medium without additives for a final 2 days.

### Fatty Acids

Stearic acid, oleic acid, or linoleic acid was all loaded onto fatty acid free BSA according to the method reported by Spector and Hoak [19]. Briefly stearic acid (0.5 g) was dissolved in chloroform (100 mL), mixed well with 10 g diatomaceous earth in a 1 liter flask. The mixture was stirred and dried under nitrogen until powder. Fatty acid free BSA (1 g) was dissolved in 100 mL DMEM without phenol red, mixed with 3 g of the stearic acid/diatomaceous earth mixture and stirred for 30 minutes. The stearic acid/BSA solution was filtered through a 0.45 µm filter, and adjusted to pH 7.4. The concentration of stearic acid in the solution was detected by use of a NEFA (non-esterified fatty acids) C kit (Wako Chemicals, Richmond, VA). The final molar ratio of stearic acid to BSA was 5 to 1, which is consistent with studies indicating 7 total fatty acid binding sites on albumin, 5 of which are considered high affinity binding site candidates [20]. Oleic and linoleic acid were loaded in the same way. All experimental data on 3T3L1 cells were confirmed using fatty acid free BSA control solutions that were subject to the same preparatory procedure described except for the fact that no fatty acid was added (controls).

### Flow cytometry analysis

After treatment, 3T3L1 cells were harvested, washed with cold phosphate buffered saline (PBS), and then resuspended in 100 µL annexin-binding buffer (50 mM HEPES, 700 mM NaCl, 12.5 mM CaCl_2_, pH 7.4). Cell density was determined, and the cells were diluted to 10^6^ cells/mL. Then 5 µL of Alex Fluo 488 annexin V and 1 µL propidium iodide (PI) were added. Cells were gently oscillated and incubated for 15 minutes at room temperature. After adding 400 µL of binding buffer to each tube, cells were kept on ice and analyzed by flow cytometry within 1 hour. Cells that stained positive for Alex Fluo 488 annexin V and negative for PI were considered to be apoptotic. Cells that stained positive for both Alex Fluo 488 annexin V and PI were considered either in the end stage of apoptosis (programmed cell death), or necrotic. Cells that stained negative for both Alex Fluo 488 annexin V and PI were considered viable and not undergoing measurable apoptosis. A BD LSR II flow cytometer from Becton Dickinson was used in all flow experiments and the data were analyzed with BD FACS Diva™ software V.6.1.3.

### Reverse transcription polymerase chain reaction (*RT-PCR*)

3T3L1 cells were treated with 50 µM stearic acid, oleic acid, linoleic acid or vehicle for 48 hours. Total RNA was extracted and purified with TRIZOL Reagent (GIBCO Invitrogen, Carlsbad, CA). The first-strand cDNA synthesis was achieved using the iScript cDNA Synthesis Kit (Bio-Rad, Hercules, CA). The PCR assay was performed in a volume of 50 µl, containing 4 µl DNA template, 45 µl Platinum PCR Supermix (Invitrogen) and 0.2 µM each specific primer. PCR was started with 3 min pre- denaturation, at 95°C, and 30 cycles of denaturation (95°C, 30 s), annealing (52°C, 30 s) and 1 min extension (72°C). The PCR products (20 µl) were analyzed by means of 1% agarose in tris-acetate ethylenediaminetetraacetic acid gel electrophoresis and visualized by ethidium bromide staining under ultraviolet; digital images were analyzed by means of a Fuji Medical System (FUJIFILM) and the bands quantified by Quantity Software (FUJIFILM). The calculated result represents the relative expression levels of target genes compared with its expression in a vehicle group after the value of the target genes was normalized to glyceraldehyde 3-phosphate dehydrogenase (GAPDH) expression levels.

The sequences of the forward and reverse mouse primers used were as follow: cIAP2 (sense 5′-CGG GAA ATT GAC CCT GCG-3′; antisense 5′-GTG CGC ACT GTG CCC TTG-3′), BAX (sense 5′-CGG CGA ATT GGA GAT GAA CTG-3′; antisense 5′-GCA AAG TAG AAG AGG GCA ACC-3′), Bcl2 (sense 5′-TAC CGT CGT GAC TTC GCA GAG-3′; antisense 5′-GGC AGG CTG AGC AGG GTC TT-3′) and GAPDH (sense 5′- CCA TCA CTG CCA CTC AGA AGA C-3′; antisense 5′-TAC CCT GAG CCA TGT AGG-3′). All pairs of primers were synthesized by Invitrogen (CA).

### Cytotoxicity assay

Lactate dehydrogenase (LD) release was measured using a cytotoxicity detection kit, according to the manufacturer's protocol (Roche Molecular Biological Co., Indianapolis, IN). After 3T3L1 cells were treated, 1 ml of cell culture medium was removed, centrifuged at 1,000 g for 5 minutes and the supernatant was assayed. Background release from culture medium alone was subtracted before reporting.

### Trypan blue staining

After treatment, 3T3L1 cells were harvested and stained with 0.4% trypan blue solution. Cells in the four corners of the grid (∼1200 cells) were counted under a conventional bright field binocular microscope. The ratio of the number of cells stained blue to the total number of cells was calculated and analyzed.

### Oil Red O staining

Cellular lipids were stained with oil red O. Briefly, identical numbers of 3T3L1 cells were placed in 6-well plates, cultured and converted to adipocytes as described above. The cells were then fixed with 4% paraformaldehyde for 30 minutes and stained with a working solution of oil red O for 5 minutes. The cell nucleus was counterstained with hematoxylin and 200 cells were counted under the microscope for each sample. The percentage of converted adipocytes was then calculated. For OD measurement, cells were stained with oil red O, the oil red O was then eluted with 1 ml of 100% isopropanol, and the OD was measured at 520 nm with a Biotek Synergy 2 Multi-Mode Microplate Reader (BioTek Instruments, Winooski, VT).

### Caspase-3 Activity Assay

Caspase-3 activity was measured using the EnzCheck Capase-3 Activity Kit #1 according to the manufacturer's instructions (Invitrogen, Carlsbad, CA). Briefly, 3T3L1 cells in a 6-well plate were washed, harvested and resuspended in 50 µl cell lysis buffer (10 mM Tris, pH 7.5, 100 mM NaCl, 1 mM EDTA, 0.2% TRITON X-100), and incubated on ice for 30 min. The lysate was then centrifuged at 5000 rpm for 5 minutes and 50 µl of supernatant was transferred to individual microplate wells. To each sample, 50 µl of substrate was added. After incubation for 30 minutes at room temperature, the fluorescence was measured with a Biotek Synergy 2 Multi-Mode Microplate Reader (BioTek Instruments, Winooski, VT), excitation/emission, 341/441. Results were quantitated using a standard curve generated for these experiments.

### Adipocyte Morphometry

Histological sections of abdominal fat were prepared and Hematoxylin & Eosin (H&E) stained. Briefly, abdominal fat tissues were fixed in 10% neutral buffered formalin (NBF) for at least 48 hours. Then the samples were processed with a VIP 1000 tissue processor through graded alcohols and xylene, and embedded into paraffin blocks. Five micron sections were cut on a Leica 2135 rotary microtome, air-dried, deparaffinized and stained with Hematoxylin & Eosin. The average size of adipocytes was measured with an Olympus BX51 system (Olympus American INC, Center Valley, Pennsylvania) Bioquant. Image Analysis software (Rtm Biometrics, Nashville, TN) was used to evaluate the H&E stained paraffin sections of fat tissue. Five representative areas were selected at a magnification of X 1.25. Each area of 0.2 mm^2^ (20–50 cells) was then measured at 10X; and the number of adipocytes in the area counted. The average adipocyte size was calculated according to the area and cell number (n = 5).

### Statistical Analysis

Data were presented as the mean +/− standard error of the mean (SEM). Overall comparisons of means were performed by one-way analysis of variance (ANOVA). A multiple comparison adjustment for the p-values and confidence limits for the differences of the least squares means was performed by the Tukey-Kramer test. For all analyses a p value of <0.05 was deemed statistically significant. Analyses were performed with SigmaStat Ver. 3.1.

## Results

### Diets, food intake and weight

**Figure 1:**
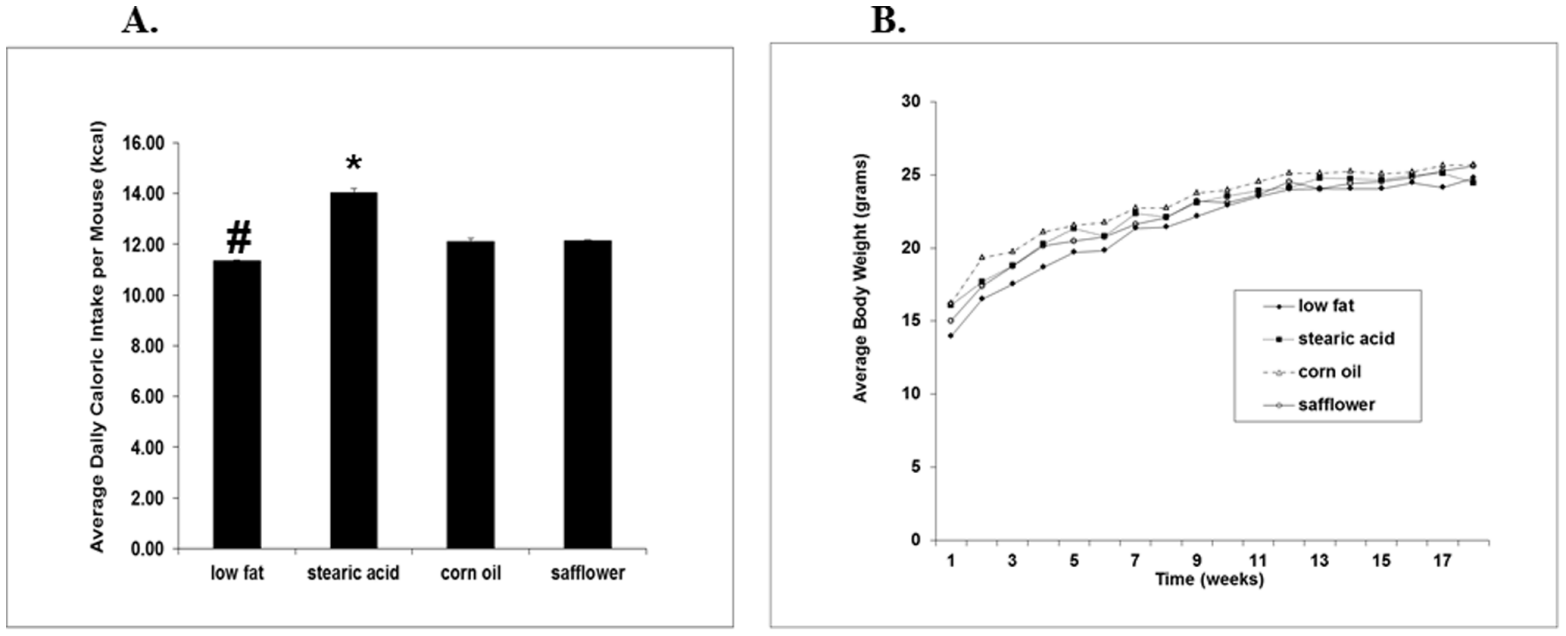
Average daily caloric intake and average weekly body weights. There were 10 mice in each diet group. (A) *, stearic acid vs. all other diets, p<0.01. #, low fat vs. all other diet groups, p<0.007. (B) There were no significant changes in body weight among the four experimental groups throughout the study. The average initial body weight was 15.3±2.6 g (p = 0.277) and the final body weight was 25.0±2.3 g (p = 0.203).

As shown in Fig.1 A, mice on the low fat diet consumed slightly fewer calories than mice on the other diets. However, despite differences in food intake, there were no significant differences in weight gain between the diets over the 18 week course of the study. (Fig. 1 B).

### Dietary stearic acid leads to a reduction of abdominal fat and total body fat (TBF)

**Figure 2:**
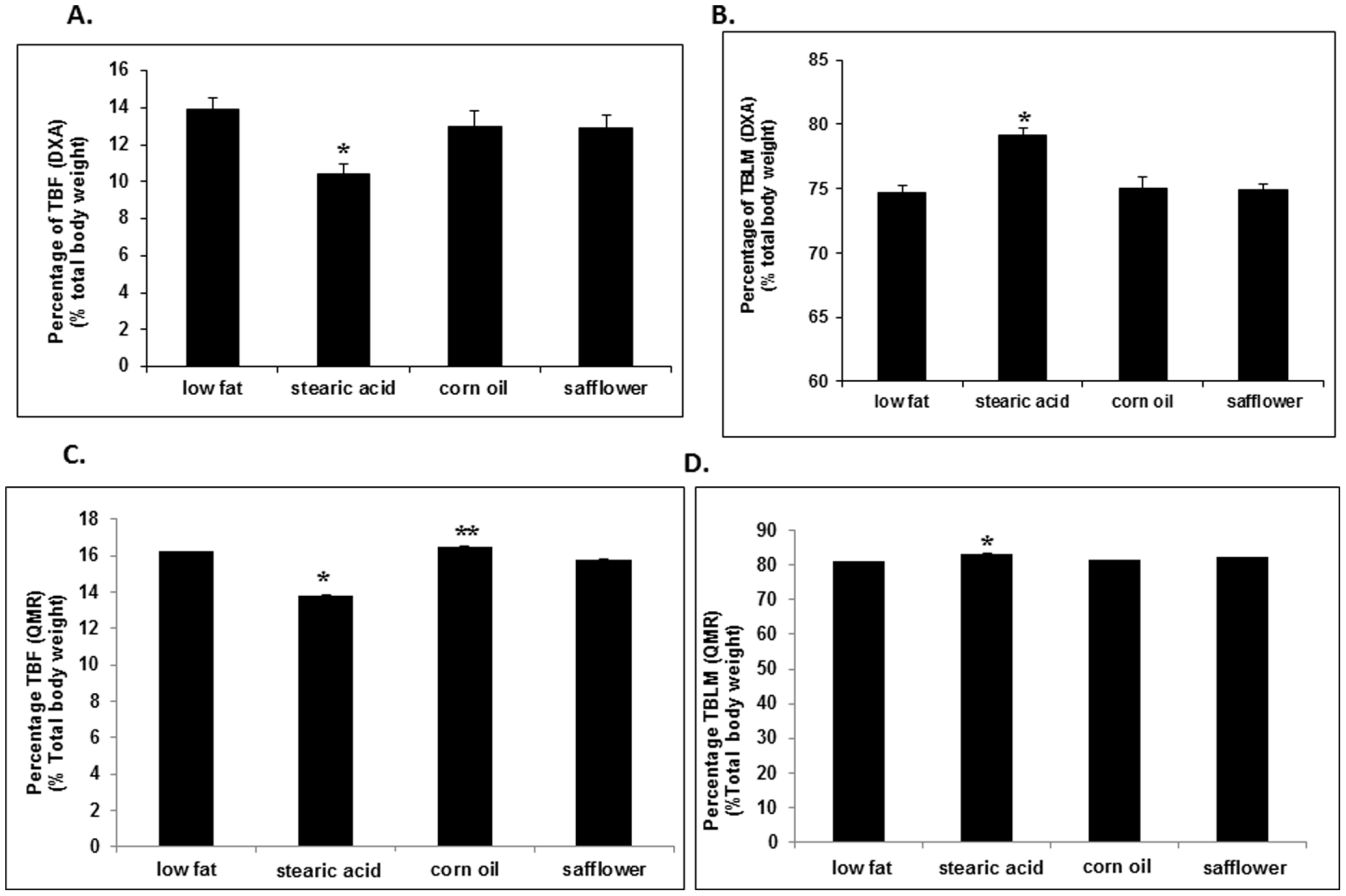
Body composition measured by DXA and QMR at week 18. Total body fat (TBF) and total body lean mass (TBLM) were assessed by DXA (A, B) and QMR (C, D), n = 10 per diet group. Mice on the stearic acid diet had a 25% decrease in TBF by DXA and 15% by QMR as compared to the low fat diet fed mice (*, p<0.009) and a 12% decrease as compared to mice on the corn oil diet by QMR **, p<0.008). Mice on the stearic acid diet also had a 4% increase in TBLM measured by DXA when compared to when compared to the other diet groups (*, p<0.01) and to the low fat diet fed mice when measured by QMR (*, p = 0.047).

The percentage of TBF decreased 25% (Fig. 2 A), while the percentage of total body lean mass (TBLM) increased 4% (Fig. 2 B) in the stearic acid diet group compared to the low fat mice when measured by DXA. QMR results confirmed the DXA results but also found that the stearic acid group had reduced TBF compared to the corn oil group (Figs. 2C-D).

Mice on the stearic acid diet also had a significantly reduced bone mineral density, 15%±1.6% SEM on average (p<0.0005), compared to all other experimental groups (data not shown for other groups) when measured by DXA.

**Figure 3:**
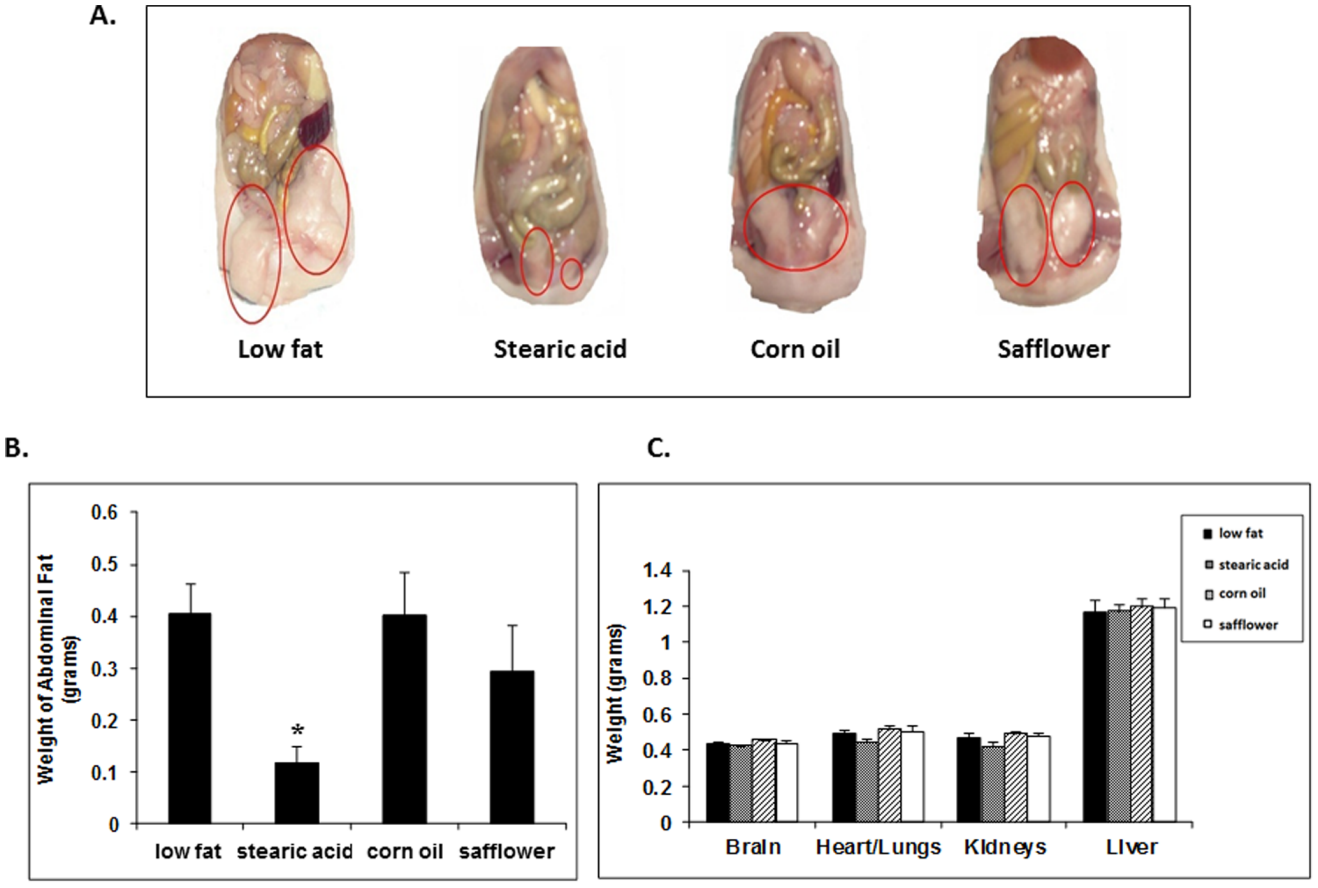
Abdominal fat and organ weight. (A) Abdominal fat images are representative of each experimental group. (B) Mice on the stearic acid diet had significantly less abdominal fat when compared to the low fat and corn oil groups (*, p<0.01, n = 10 per diet group). (C) There was no difference in the weight of heart/lungs, liver, kidney or brain between any of the dietary groups.

As shown in Fig. 3, A and B, abdominal fat was found to be decreased by 67% in the stearic acid diet group when compared to the low fat diet group. The weights of brain, heart/lungs, kidneys and liver were not significantly different among the different dietary groups (Fig. 3C). Kidney and liver histological sections were prepared, stained with H&E (see Figs. S1, S2 in Data S1) and evaluated by two experienced pathologists who found no meaningful pathological changes.

**Figure 4:**
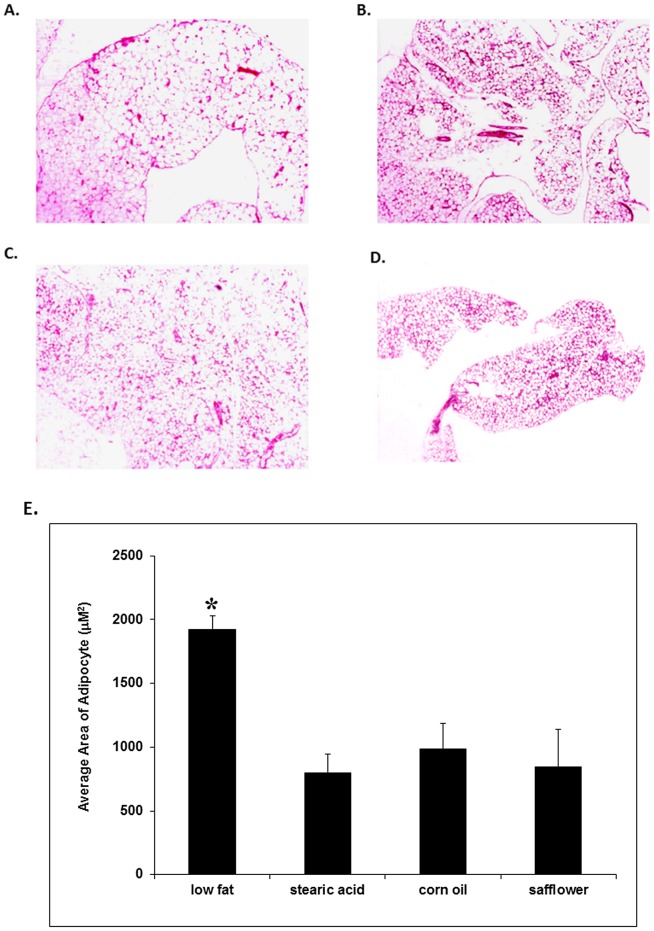
The size of abdominal adipocytes in dietary groups. Representative images of histopathologic sections of abdominal fat from mice fed: a low fat diet (A), corn oil diet (B), safflower oil diet (C) or stearic acid diet (D) All images are at the same low power (25x) magnification. (E) Mice on the low fat diet had significantly larger adipocytes as compared to all other groups (*, p<0.01).

As shown in Fig. 4A-E, abdominal adipocytes from mice on the low fat diet were on average 119% larger as compared to the other dietary groups when measured by morphometric analysis.

### The effect of dietary stearic acid on serum glucose, insulin, and inflammatory cytokines (Figure 5)

**Figure 5:**
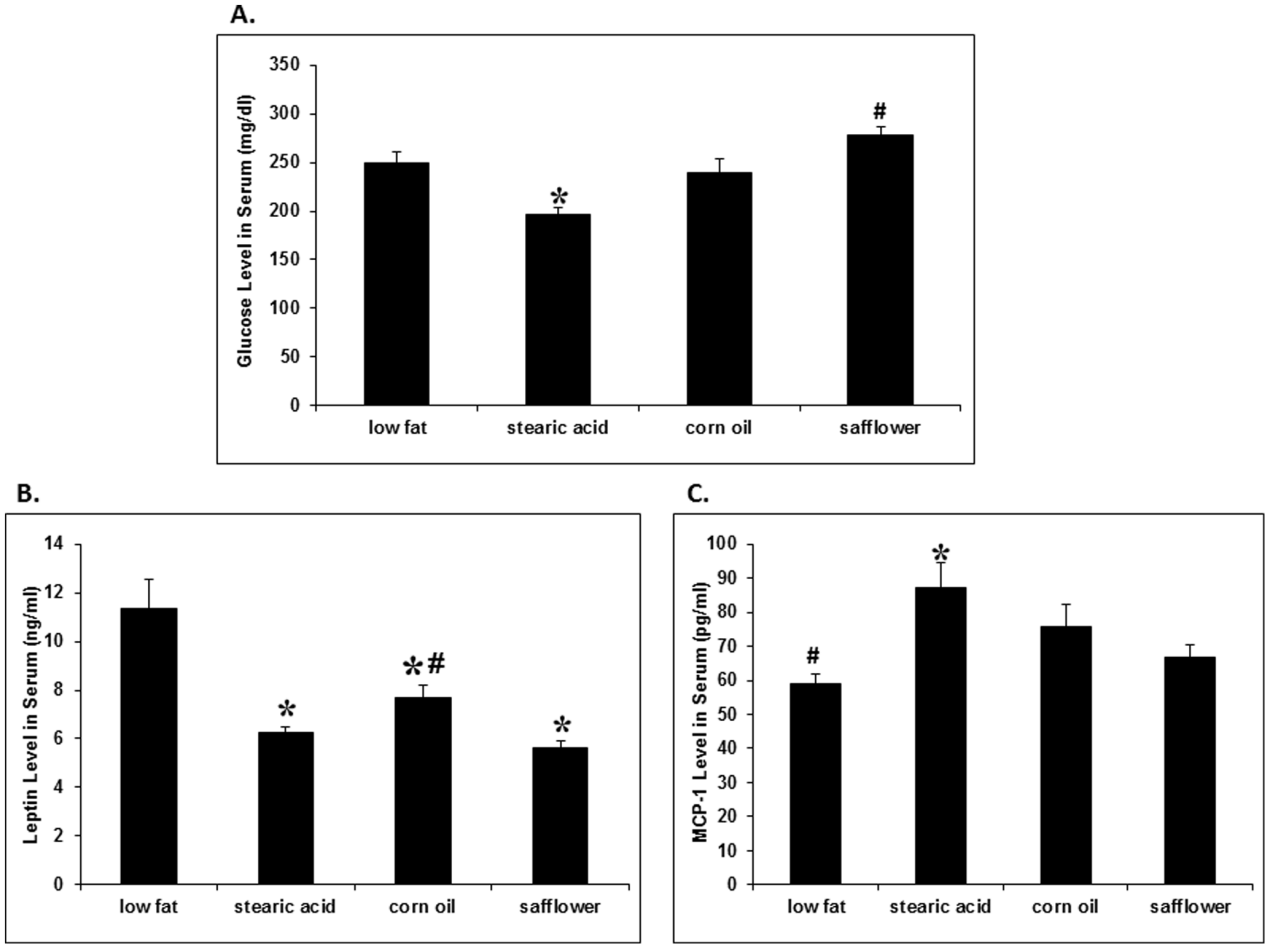
Serum analyte analysis. N = 10 per diet group. (A) Mice on the stearic acid diet had significantly reduced serum glucose compared to all other experimental groups (*, stearic acid vs. low fat, p = 0.006; corn oil, p = 0.039; safflower oil, p<0.001). Mice on the corn oil diet also had a significantly reduced level of glucose as compared to the safflower group (#, p = 0.034). (B) Mice on the high fat diets had significantly reduced levels of leptin as compared to the low fat group (*, low fat vs. stearic acid, p<0.001; corn oil, p = 0.014; safflower oil, p<0.001). Mice on the stearic acid and safflower oil diets also had a significantly lower level of leptin when compared to the corn oil group (#, p = 0.015 and 0.003, respectively). (C) Mice on the stearic acid diet had a significantly increased level of MCP-1 when compared to the low fat and safflower oil groups (*, p = 0.003 and 0.019, respectively). Mice on the low fat diet also had a significantly reduced level of MCP-1 when compared to the corn oil group (#, p = 0.032).

Serum glucose was decreased in mice on the stearic acid diet as compared to the other diets on average by 22%±3 (SEM). Serum leptin was significantly decreased in all high fat diets as compared to the low fat diet. In addition mice on the stearic acid and safflower oil diets had significantly lower concentrations of leptin as compared to the corn oil (14% and 18%, respectively). Serum monocyte chemotactic protein-1 (MCP-1) was significantly increased in the stearic acid diet group as compared to the low fat and safflower oil groups (35% and 26%, respectively). In addition, the low fat diet had a reduced MCP-1 as compared to the corn oil group by 17%. The serum concentrations of insulin, IL-6 and adiponectin were the same among the different diet groups (p = 0.46, p = 0.46, p = 0.074, respectively, data not shown).

### The effect of stearic acid on the differentiation of 3T3L1 cells

In order to investigate the possible mechanism of fat reduction caused by stearic acid, we examined the direct effect of stearic acid on the differentiation of mouse 3T3L1 preadipocyte cells. 3T3L1 cells were treated with 50 µM stearic acid during the differentiation process and subsequently stained with oil red O to determine the amount of fat accumulation. Fat cell differentiation, as assessed by the ratio of manually counted differentiated to undifferentiated cells or by the amount of fat accumulation in cells as measured by oil red O was not affected by stearic acid (see Fig. S3 in Data S1).

### The effect of stearic acid on cell death of adipocytes and preadipocytes

Induction of programmed cell death (apoptosis) by adipocytes through direct contact with stearic acid may be a driving force for body fat reduction. We first converted 3T3L1 cells into adipocytes and then treated them with 50 µM of stearic acid, oleic acid or linoleic acid, and examined the percentage of apoptotic and necrotic cells. We found that stearic acid, oleic acid and linoleic acid had no effect on the percentage of injured, apoptotic or necrotic cells (see Fig. S4 in Data S1).

**Figure 6:**
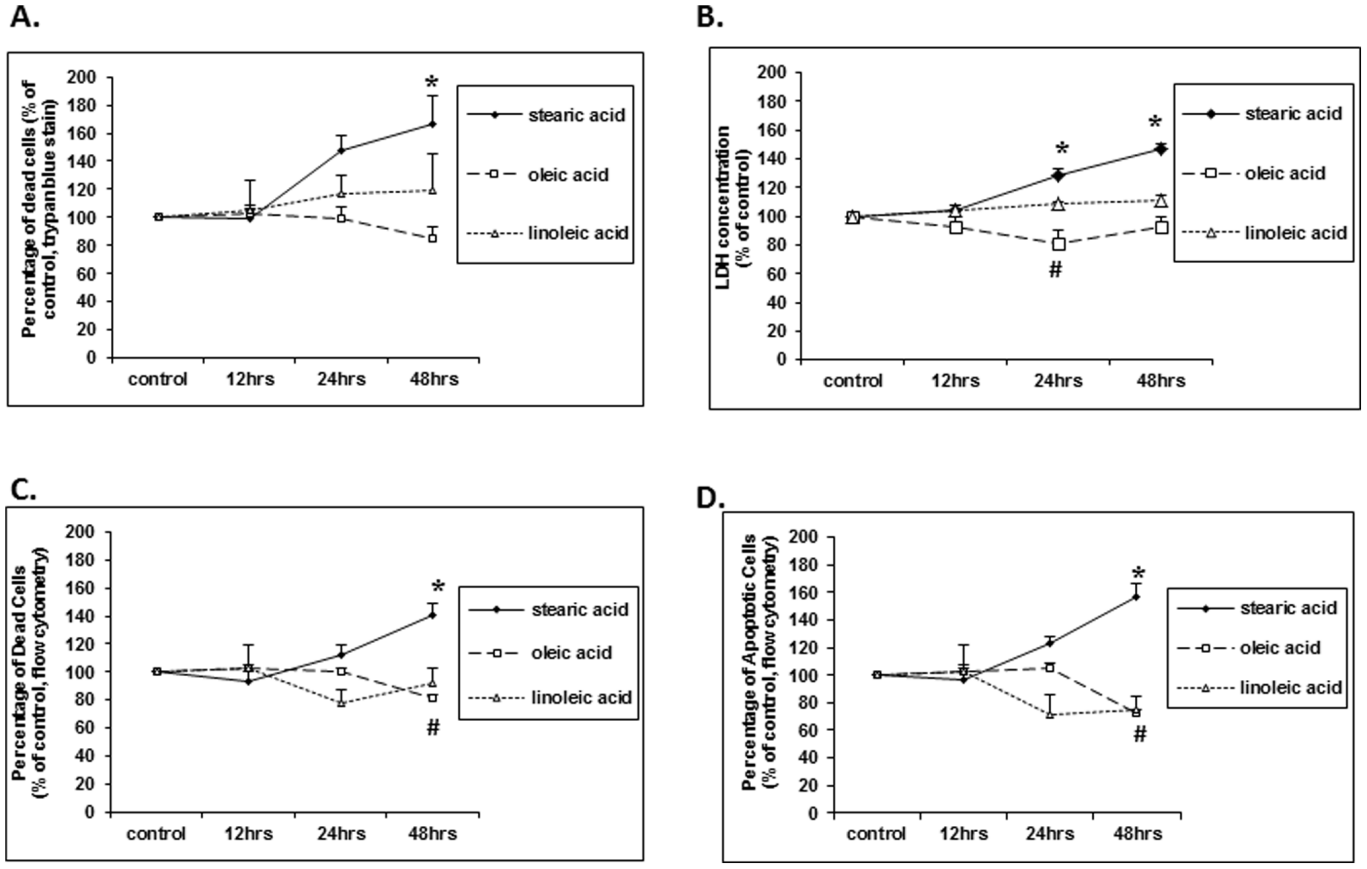
Effects of 50 µM stearic acid, oleic acid and linoleic acid on cell death and apoptosis of 3T3L1 preadipocytes. (A) Trypan blue staining showed that the percentage of dead cells was significantly increased after a 48 hour treatment with stearic acid (*, p<0.01, compared to control, n = 3). In contrast, oleic acid or linoleic acid had no significant changes over time (p>0.05, n = 3). (B) Cytotoxicity was significantly increased after 24 hours of treatment with stearic acid (*, p<0.01, compared to control, n = 5). However, it was significantly decreased with 24 hours of oleic acid treatment (#, p<0.01, compared to control, n = 4). Cytotoxicity did not change significantly with linoleic acid treatment (p>0.05, n = 4). (C) Flow cytometry revealed an increase in dead cells after 48 hours of treatment with stearic acid (*, p<0.01, compared to control, n = 4), while oleic acid significantly decreased the number of dead cells at 48 hours (#, p<0.01, compared to control, n = 4). In contrast, linoleic acid had no significant effects over time (n = 4). (D) Apoptotic cells were significantly increased with stearic acid treatment (*, p<0.01, compared to control, n = 4) and decreased with oleic acid (#, p<0.01, compared to control, n = 4). No significant changes were observed with linoleic acid treatment (p>0.05, n = 4).

To determine whether stearic acid has a direct effect on preadipocytes, we used undifferentiated 3T3L1 cells. We showed that stearic acid increased preadipocyte cytotoxicity as measured by trypan blue exclusion (Fig. 6A). In contrast oleic acid decreased cell injury as measured by lactate dehydrogenase in the media (Fig. 6B), and when flow cytometry was used to detect these cells (Fig. 6C). Measuring apoptosis by flow cytometry we found that stearic acid increased apoptosis of preadipocytes while oleic acid decreased apoptosis (Fig. 6D).

**Figure 7:**
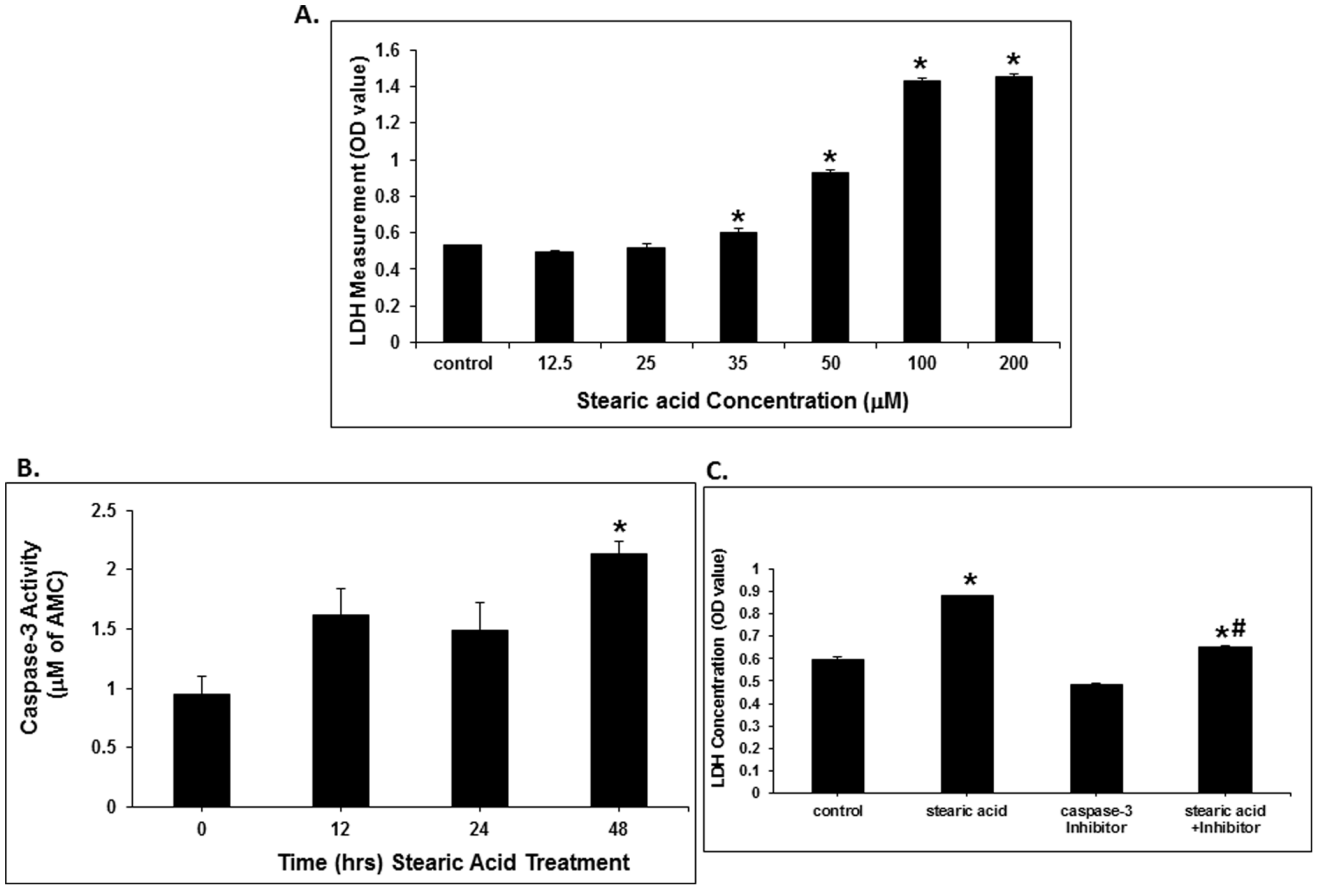
Cytotoxicity effect of stearic acid on 3T3L1 preadipocytes. (A) Lactate dehydrogenase concentrations increased significantly when the dose of stearic acid was over 35 µM after 48 hours of treatment, and peaked at 100–200 µM (*, p<0.01, compared with the control group, n = 4). (B) Measurement of caspase-3 activity showed stearic acid (50 µM) increased caspase-3 activity of the cultured preadipocytes after 48 hours' treatment (*, p<0.05, compared to control, n = 4). (C). When preadipocytes were pretreated with a specific caspase-3 inhibitor for 4 hours, and then treated with stearic acid (50 µM), the stearic acid induced cytotoxicity was partially inhibited (*, p<0.01, compared with the control group; #, p<0.01, n = 3).

**Figure 8:**
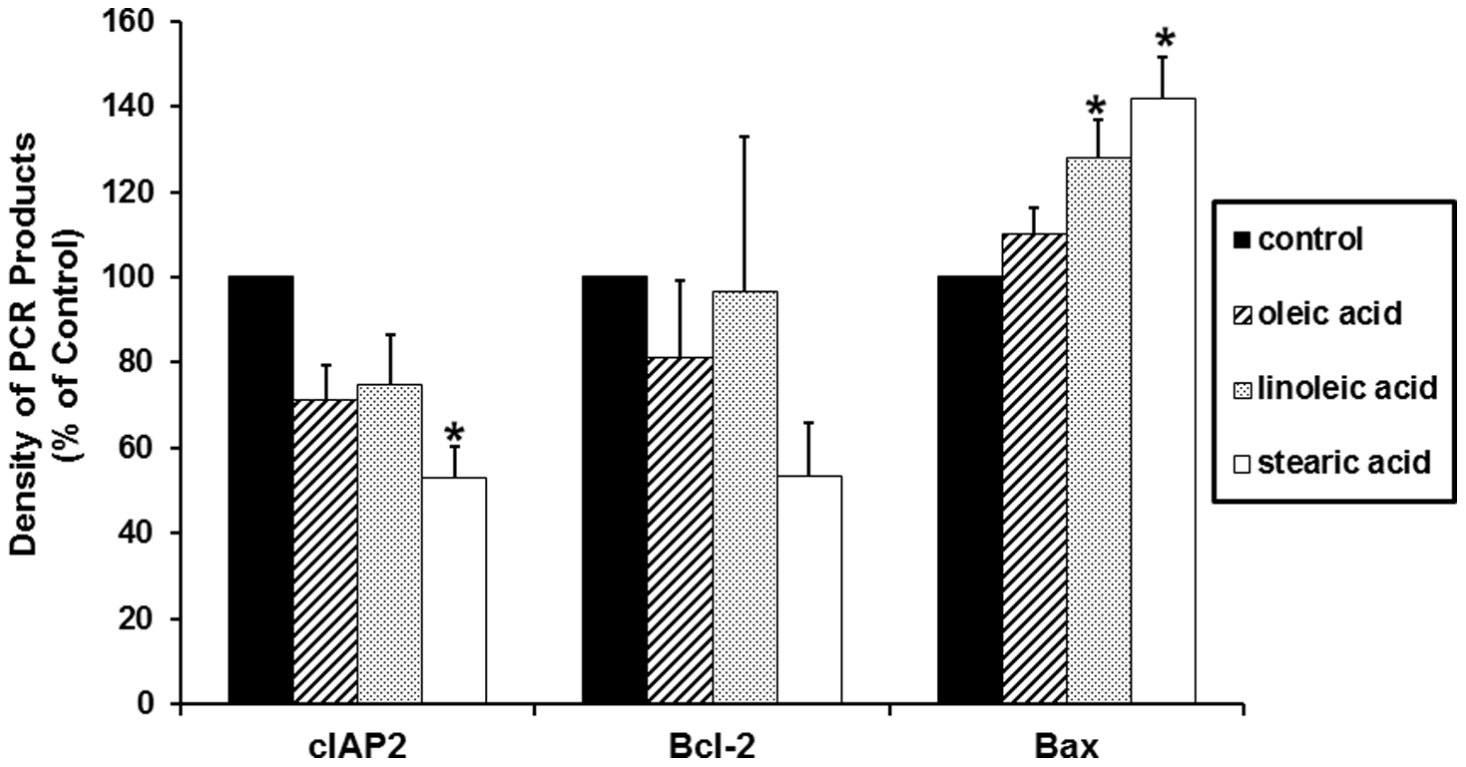
Effects of 50 µM stearic acid, oleic acid or linoleic acid on gene expression in 3T3L1 preadipocytes; Control cells were treated the same as fatty acid treated cells except they were treated with fatty acid free BSA. Cells were treated for 48 hours. These data showed that stearic acid decreased the expression of cIAP2 and Bcl2 (although only cIAP2 was significant, n = 6 replicates * p<0.01), which encodes antiapoptotic proteins, and increased Bax gene expression, which encodes for a proapoptotic protein, when compared to control cells n = 6 replicates, (* p<0.01). Linoleic acid also increased expression of Bax compared to control cells (n = 6 replicates, * p<0.01).

To verify the inhibitory effect of stearic acid on preadipocytes, we treated cultured preadipocytes for 48 hours with different doses of stearic acid. As shown in Fig. 7 A, stearic acid-induced cytotoxicity was dose-dependent beginning at 35 µM and reaching a maximum at 100 µM. In order to confirm an apoptotic effect of stearic acid on preadipocytes, we measured caspase-3 activity. As shown in Fig. 7 B, caspase-3 activity increases significantly after 48 hours of stearic acid treatment, which is consistent with the flow cytometry results. When preadipocytes were pretreated with a caspase-3 inhibitor, as shown in Fig. 7 C, stearic acid-induced cytotoxicity was partially inhibited. We also investigated the expression of apoptosis related genes in preadipocytes. When 3T3L1 preadipocytes were treated with 50 µM stearic acid, oleic acid or linoleic acid for 48 hours, as shown in Fig. 8, stearic acid decreased the expression of cIAP2 and Bcl2, and increased Bax gene expression when compared to control cells (p<0.01) (Fig. 8, and Fig. S5 in Data S1).

In summary, we have shown for the first time that dietary stearic acid leads to a reduction in visceral fat when compared to both a low fat control diet and a corn oil diet. This reduction in visceral fat was accompanied by reduced serum glucose and leptin but no apparent morphologic adverse effects on the liver or kidney. In addition, we showed that stearic acid, but not oleic acid or linoleic acid, caused injury and cell death via apoptosis in preadipocytes. In contrast, stearic acid had no significant effect on fat cell differentiation or on fully differentiated adipocytes.

## Discussion

Our main finding was that dietary stearic acid leads to dramatically reduced visceral adipose tissue (VAT). Our data also indicated that total body fat was reduced by 25% compared to the low fat diet group when standardized to total body weight. Specifically, when VAT was normalized to total body weight, the stearic acid group had 67%±4% (SEM) less VAT as compared to the other diet groups. A conservative estimate of the normal percentage of VAT compared to total mouse fat is 60% [21] and a 67% reduction in VAT would reduce total body fat by 40% which is more than enough to account for the 25% loss of total body fat measured by DXA (which was also standardized to total body weight). Thus our data strongly point to VAT as being the primary fat depot being affected by dietary stearic acid. However we did not measure possible changes in other fat depots and cannot rule out rule out the possibility that fat was lost or gained from other depots. Also we don't know the long term effects of dramatically reducing VAT. Nevertheless it is clear that excess VAT is not healthy and a diet that reduces VAT in a setting of excess VAT may be beneficial.

Is reduction in VAT associated with reduced inflammation and improved glucose handling? While neither inflammation nor glucose control was measured directly, histopathology of liver and kidney sections indicated that none of the diets tested were associated with morphologic changes associated with inflammation (i.e. infiltration of leukocytes). Serum markers of inflammation were mixed with IL-6 demonstrating no change among the diets while MCP-1 was increased. MCP-1 or chemokine ligand 2 (CCL2) has chemoattractant properties for immunological cells honing them to sites of injury and resulting in inflammatory responses. Interestingly VAT expresses more MCP- 1 than SAT in obese individuals [22] so it is not clear why MCP-1 is increased in mice on the stearic acid diet with reduced VAT. MCP-1 is also produced by bone cells such as osteoblasts and osteoclasts as well as smooth muscle cells and endothelial cells so it may be coming from one or more of these sources. In fact we did note that bone mineral density was reduced in mice on the stearic acid diet possibly indicating increased osteoclast activity. Interestingly MCP-1 appears to play a role in tumor associated increased production of osteoclasts and bone resorption in prostate cancer [23]. In terms of breast cancer, increased serum concentrations of MCP-1 were associated with less advanced disease [24]. Nevertheless the reason for increased MCP-1 must be determined in future studies which will also include a more detailed investigation for the presence of inflammation.

Serum glucose was decreased in the stearic acid diet group compared to other diets while serum insulin and adiponectin levels did not change significantly, which is not definitive but certainly consistent with improved insulin sensitivity. Serum leptin was decreased in the dietary stearic acid group. Leptin is released from adipocytes and suppresses appetite via receptors in the hypothalamus. It is possible that the observed increased food intake in the dietary stearic acid group may be due to decreased leptin. It should also be noted that increased leptin has been associated with obesity as well as promoting breast cancer growth and increasing angiogenesis [the formation of new blood vessels] [25]. Thus the decreases in leptin and glucose may be beneficial effects especially when combined with no difference in overall body weight and insulin, reduced VAT and increased total body lean mass were seen in mice on the stearic acid diet. Nevertheless, these results are not definitive but rather provide rationale for a more in depth study.

How does stearic acid reduce VAT? Data presented herein indicate that stearic acid preferentially causes apoptosis of preadipocytes but not mature adipocytes. We also demonstrate that stearic acid does not affect adipocyte differentiation. Others have found that palmitate, a 16 carbon saturated fatty acid, also induces apoptosis in 3T3-L1 preadipocytes likely via endoplasmic reticulum stress [26]. However, this effect of palmitate was not seen at 100 µM and was first noted at 250 µM palmitate. Most of the experiments were done at 250 µM and 500 µM palmitate which are 5 to 10 times as high as those used in our experiments, respectively. Other studies have indicated that long chain saturated fatty acids, such as stearic acid and palmitate, are capable of causing apoptosis in various cell types [16], [17]. However these studies almost uniformly used concentrations that were ≥100 µM. This minimum concentration of 100 µM used previously is arguably not physiologic and double what was used in these studies. While a more detailed discussion of this issue is not warranted here, it is clear that stearic acid can cause apoptosis in cells; however, this property is very dependent on stearic acid concentration, time of exposure and cell type. Stearic acid normally constitutes approximately 10% of circulating free or non-esterified fatty acids in humans [18]. If one considers ∼500 µM of total free or non-esterified fatty acids to be at the high end of “normal” for the U.S. population then 50 µM stearic acid is approximately at the high end of normal. Unfortunately, the authors did not demonstrate a specific effect on preadipocytes although the authors do mention palmitate has demonstrated a similar effect in various cell types [16], [17]. In addition, the fact that these effects were not seen at 100 µM, suggest it may be a different effect compared to stearic acid.

Another study indicated a diet high in stearic acid is lipogenic and resulted in liver fat accumulation and increased body weight at 8 weeks [27]. We did not see these same effects, however there were experimental differences in the diets in that they used tristearin which is a glycerol ester of stearic acid (i.e. stearic acid containing triglycerides) while we used a purified form of stearic acid. Interestingly, when stearoyl- CoA desaturase-1 (SCD-1) deficient mice were the recipients, this lipogenic effect was not seen while both adult adipose tissue and body weight were decreased. It is possible that stearic acid is decreasing SCD-1 expression unlike tristearin which increased SCD-1 expression. However based on the widespread presence of SCD-1 in the liver and different adipose depots and the fact that decreased leptin concentrations increase SCD-1 we also think it unlikely that dietary stearic acid, which shows a selective effect on VAT and decreases leptin concentrations, is working through this same proposed mechanism. Importantly, dietary stearic acid per se is not preferentially incorporated into triglycerides, unlike palmitate, but is largely incorporated into phospholipids [28].

How does stearic acid cause apoptosis of preadipocytes? Others have demonstrated that preadipocytes from different fat depots have different gene expression patterns in humans [29]. Of note, the inhibitor of apoptosis (IAP) family of proteins, as their name suggests, inhibit apoptosis while family member cIAP2 is overexpressed in visceral preadipocytes compared to subcutaneous preadipocytes [30]. This suggests that it may be more important to the host for the survival of its visceral preadipocytes as compared to its subcutaneous preadipocytes. Importantly, cIAP2 is also expressed in various mouse tissues including mouse embroyonic fibroblasts [31] although to our knowledge no one has looked at mouse preadipocytes. Since the selective effect of dietary stearic acid on preadipocytes appears to be working through apoptosis, it is likely that an apoptosis related gene or genes are involved. For example, it is possible that dietary stearic acid inhibits the expression of cIAP2 in preadipocytes but because visceral preadipocytes are more dependent on cIAP2 for survival they are selectively reduced. Our gene expression data are consistent with this hypothesis. In our study, dietary stearic acid was initiated in young, only 5 weeks old, mice so it is possible that a reduction of preadipocytes by stearic acid also slows down further visceral fat accumulation accounting for the differences in visceral fat between the dietary groups. The precise mechanism of how dietary stearic acid selectively induces apoptosis of preadipocytes *in vitro* but not mature adipocytes remains to be determined. Interestingly, stearic acid has been shown to have a selective apoptosis effect on breast cancer cells [32]. Further studies are needed to determine how stearic acid is causing apoptosis in preadipocytes. It is hoped that these studies lead to a specific target molecule to reduce adipocytes. Another consideration is whether dietary stearic acid could be used to reduce VAT? The answer is not clear. The optimum concentration of dietary stearic acid needs to be determined as well as more detailed toxicity studies. In human studies, dietary stearic acid does not increase total serum cholesterol, unlike palmitate, nor does it increase low density lipoprotein cholesterol [LDLc] the “bad” cholesterol [33]. In addition, dietary stearic acid did not adversely affect insulin action [34], was not thrombogenic [35], and did not affect blood pressure [36] in human studies.

In terms of stearic acid and breast cancer, cell culture and animal studies indicate a beneficial inhibitory effect of stearic acid on breast cancer cell growth [31], tumor growth [31], [37], carcinogenesis [14] and metastasis [13]. Thus, it is unlikely that dietary stearic acid will promote breast cancer. Previous mechanisms elucidated for a direct apoptotic effect of stearic acid on breast cancer cells involve stearic acid incorporation into diacylglycerol, activation of protein kinase C [32] and the inhibition of Rho [14]. Again, it is important to restate that the effects of stearic acid are dependent on cell type as well as concentration. Thus, it is difficult to relate the effects of stearic acid on one cell type to the other. Nevertheless, overall these studies do not rule out the possibility of some form of dietary stearic acid being useful to reduce adipose tissue. Interestingly the use of dietary stearic acid to replace *trans*-fatty acids in foods that require solid fats has been suggested to provide beneficial effects on low density lipoprotein cholesterol (LDLc) which is the primary target for cardiovascular risk reduction [38].

While we have focused this discussion on stearic acid, visceral fat and breast cancer it is likely that reducing excess visceral fat may also be very beneficial for type 2 diabetes, the metabolic syndrome, cardiovascular disease and possibly other disease states. To this end it will be very important to confirm our findings in non-nude mice, rats and in the long term, hopefully, in humans.

Limitations of this study are that the corn oil and safflower oil diets contained fatty acids largely in the form of triglycerides while the stearic acid diet used non- esterified stearic acid. Also we did not prove that dietary stearic acid per se caused the reduction in visceral adipose tissue.

In summary, we have shown that dietary stearic acid leads to the reduction of visceral fat as well as lowering blood glucose and leptin concentrations. We also have demonstrated a specific effect of stearic acid causing apoptosis, likely by increasing proapoptotic molecules such as BAX and decreasing antiapoptotic molecules such as cIAP2, in preadipocytes but not mature adipocytes. These studies may lead to one or more targets to selectively reduce VAT and suggest that further studies to determine the effects of dietary stearic acid on CVD, diabetes and the metabolic syndrome as well as certain cancers may be indicated.

## Supporting Information

Data S1 **Supporting Figures S1-S5. Figure S1. The histopathology of kidneys.** Representative H&E stained sections of kidneys from mice fed either a low fat diet (A), corn oil diet (B), safflower oil diet (C) or stearic acid diet (D). Three kidneys per diet were examined and all kidneys examined irrespective of diet were without significant histopathologic abnormalities. **Figure S2. Liver histopathology.** Representative H&E stained sections of liver from mice fed either a low fat diet (A), corn oil diet (B), safflower oil diet (C) or stearic diet (D). Three livers per diet were examined and all livers examined irrespective of diet were without significant histopathologic abnormalities. **Figure S3. Effect of diets on 3T3L1 cell differentiation.** Representative oil red O and hematoxylin stained control (A) and stearic acid (50 µM) treated 3T3L1 (B) cells. The ratio of differentiated to undifferentiated adipocytes was not significantly different in the two experimental groups (p = 0.477, n = 6) (C). Oil red O was eluted from the cells and the OD value was measured. Again no difference was observed between the stearic acid and the control group (p = 0.149, n = 6) (D). **Figure S4. Effects of 50 µM stearic acid, oleic acid or linoleic acid on necrosis and apoptosis of differentiated 3T3L1 adipocytes.** Trypan blue stain was used to detect cell death and cytotoxicity was assessed by measurement of lactate dehydrogenase (LD) concentrations in the medium. Flow cytometry was used to investigate the necrosis and apoptosis. (A) The trypan blue stain showed that there were no significant changes in the percentage of dead cells when adipocytes were treated with stearic acid, oleic acid or linoleic acid throughout the study (p>0.05, n = 3). (B) Cytotoxicity detection similarly showed no significant changes among the three experimental treatment groups (p>0.05, n = 4). (C) There were no significant changes in the percentage of dead cells detected by flow cytometry when adipocytes were treated with stearic acid, oleic acid or linoleic acid (p>0.05, n = 4). (D) Similarly, flow cytometry revealed no significant changes in the percentage of apoptotic cells among the three experimental treatment groups (p>0.05, n = 4). **Figure S5. Effects of 50 µM stearic acid, oleic acid or linoleic acid on gene expression in 3T3L1 preadipocytes; C, control; O, oleic acid; L, linoleic acid; S, stearic acid.** Control cells were treated identical to fatty acid treated cells except they were treated with fatty acid free BSA (see methods under Fatty Acids). Cells were treated for 48 hours. These data showed that stearic acid decreased the expression of cIAP2 and Bcl2 (although only cIAP2 was significant (n = 6 replicates, p<0.01), which encodes antiapoptotic proteins, and increased Bax gene expression, which encodes for a proapoptotic protein, when compared to control cells. Linoleic acid also increased expression of Bax compared to control cells (n = 6 replicates, p<0.01). These data are consistent with our apoptosis flow cytometry data and raise the possibility of a cIAP2 mediated mechanism of action for stearic acid induced apoptosis of preadipocytes.
(DOCX)
