# Supplementary material for: Dietary Stearic Acid Leads to a Reduction of Visceral Adipose Tissue in Athymic Nude Mice
Source: PLoS One. 2014 Sep 15;9(9):e104083. doi: 10.1371/journal.pone.0104083 (PMC4164353; doi:10.1371/journal.pone.0104083)
Supplement: Data S1 — Supporting Figures S1-S5. Figure S1. The histopathology of kidneys. Representative H&E stained sections of kidneys from mice fed either a low fat diet (A), corn oil diet (B), safflower oil diet (C) or stearic acid diet (D). Three kidneys per diet were examined and all kidneys examined irrespective of diet were without significant histopathologic abnormalities. Figure S2. Liver histopathology. Representative H&E stained sections of liver from mice fed either a low fat diet (A), corn oil diet (B), safflower oil diet (C) or stearic diet (D). Three livers per diet were examined and all livers examined irrespective of diet were without significant histopathologic abnormalities. Figure S3. Effect of diets on 3T3L1 cell differentiation. Representative oil red O and hematoxylin stained control (A) and stearic acid (50 µM) treated 3T3L1 (B) cells. The ratio of differentiated to undifferentiated adipocytes was not significantly different in the two experimental groups (p = 0.477, n = 6) (C). Oil red O was eluted from the cells and the OD value was measured. Again no difference was observed between the stearic acid and the control group (p = 0.149, n = 6) (D). Figure S4. Effects of 50 µM stearic acid, oleic acid or linoleic acid on necrosis and apoptosis of differentiated 3T3L1 adipocytes. Trypan blue stain was used to detect cell death and cytotoxicity was assessed by measurement of lactate dehydrogenase (LD) concentrations in the medium. Flow cytometry was used to investigate the necrosis and apoptosis. (A) The trypan blue stain showed that there were no significant changes in the percentage of dead cells when adipocytes were treated with stearic acid, oleic acid or linoleic acid throughout the study (p>0.05, n = 3). (B) Cytotoxicity detection similarly showed no significant changes among the three experimental treatment groups (p>0.05, n = 4). (C) There were no significant changes in the percentage of dead cells detected by flow cytometry when adipocytes were treated with s [file pone.0104083.s001.docx]

**Supplemental Data:**


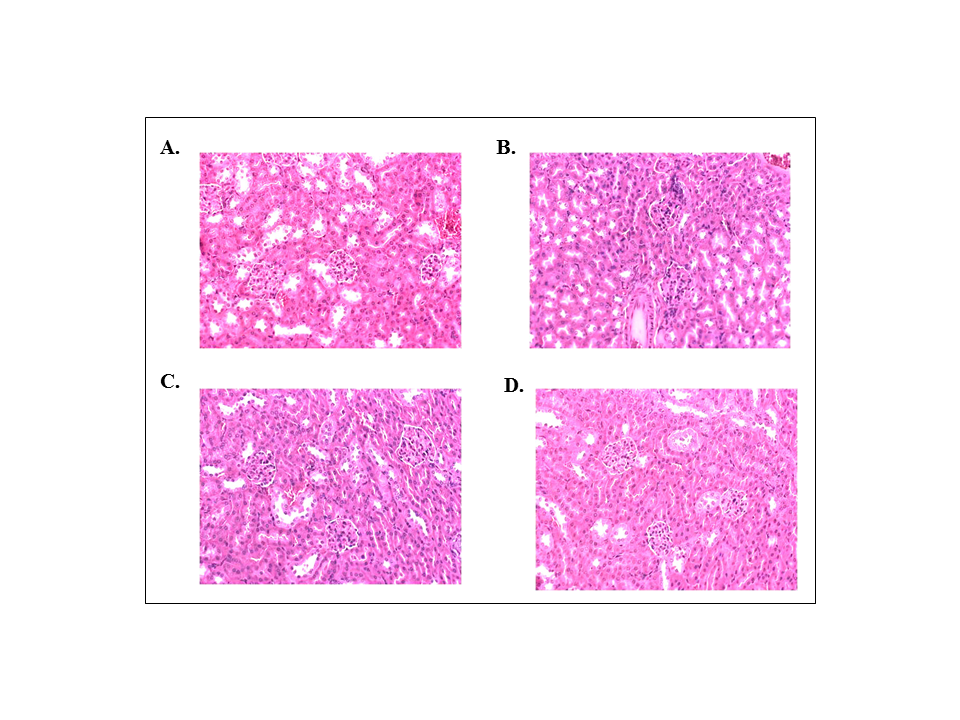


Figure S1. The histopathology of kidneys. Representative H&E stained sections of kidneys from mice fed either a low fat diet (A), corn oil diet (B), safflower oil diet (C) or stearic acid diet (D). Three kidneys per diet were examined and all kidneys examined irrespective of diet were without significant histopathologic abnormalities.


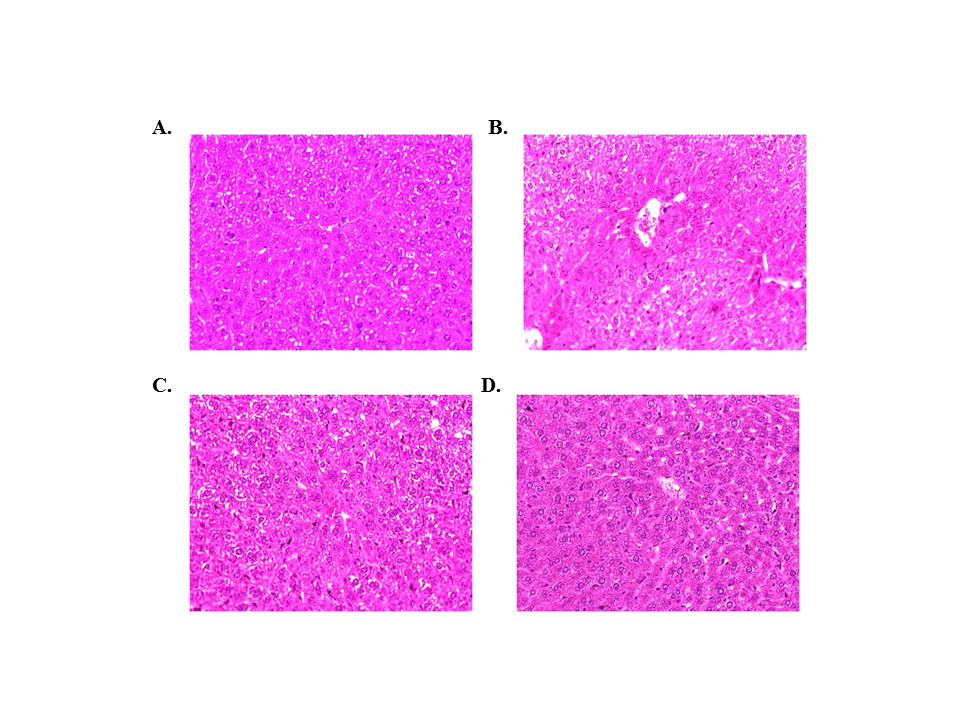


Figure S2. Liver histopathology. Representative H&E stained sections of liver from mice fed either a low fat diet (A), corn oil diet (B), safflower oil diet (C) or stearic diet (D). Three livers per diet were examined and all livers examined irrespective of diet were without significant histopathologic abnormalities.


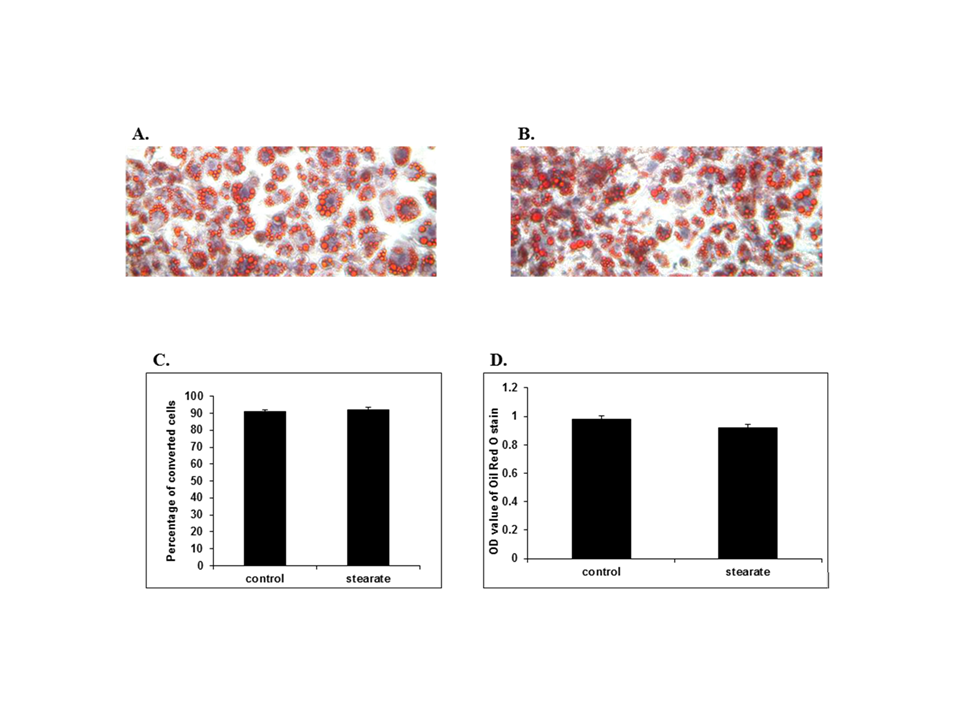


Figure S3. Effect of diets on 3T3L1 cell differentiation. Representative oil red O and hematoxylin stained control (A) and stearic acid (50 µM) treated 3T3L1 (B) cells. The ratio of differentiated to undifferentiated adipocytes was not significantly different in the two experimental groups (p=0.477, n=6) (C). Oil red O was eluted from the cells and the OD value was measured. Again no difference was observed between the stearic acid and the control group (p=0.149, n=6) (D).


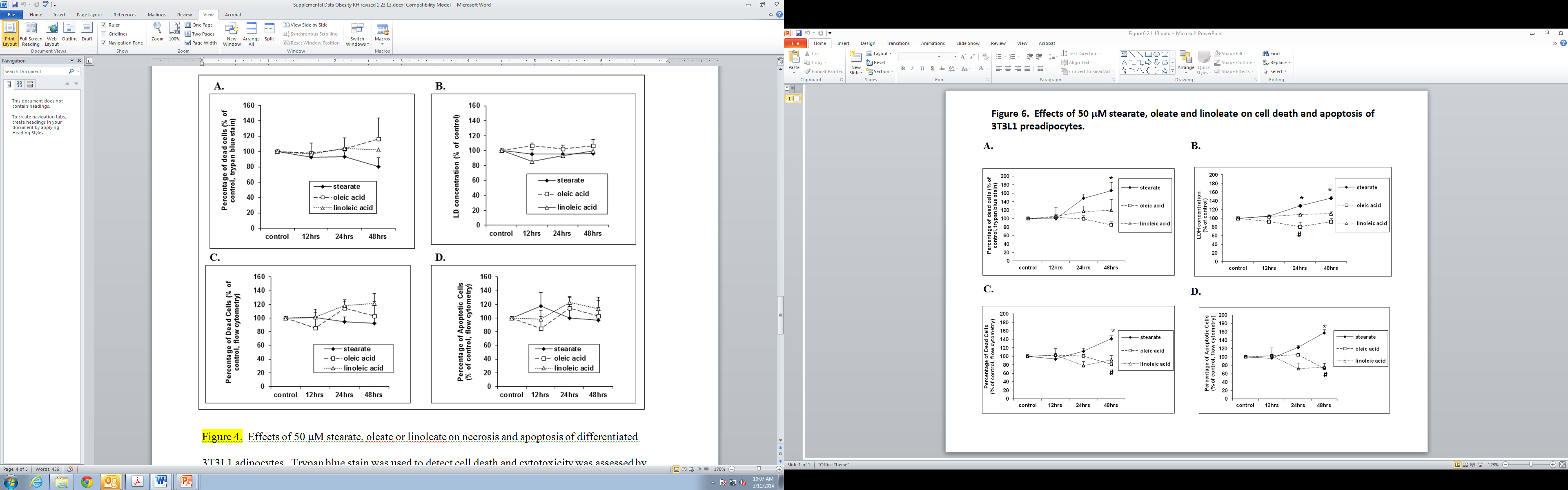


**stearic acid**

**stearic acid**

**stearic acid**

**stearic acid**

Figure S4. Effects of 50 µM stearic acid, oleic acid or linoleic acid on necrosis and apoptosis of differentiated 3T3L1 adipocytes. Trypan blue stain was used to detect cell death and cytotoxicity was assessed by measurement of lactate dehydrogenase (LD) concentrations in the medium. Flow cytometry was used to investigate the necrosis and apoptosis. (A) The trypan blue stain showed that there were no significant changes in the percentage of dead cells when adipocytes were treated with stearic acid, oleic acid or linoleic acid throughout the study (p>0.05, n=3). (B) Cytotoxicity detection similarly showed no significant changes among the three experimental treatment groups (p>0.05, n=4). (C) There were no significant changes in the percentage of dead cells detected by flow cytometry when adipocytes were treated with stearic acid, oleic acid or linoleic acid (p>0.05, n=4). (D) Similarly, flow cytometry revealed no significant changes in the percentage of apoptotic cells among the three experimental treatment groups (p>0.05, n=4).


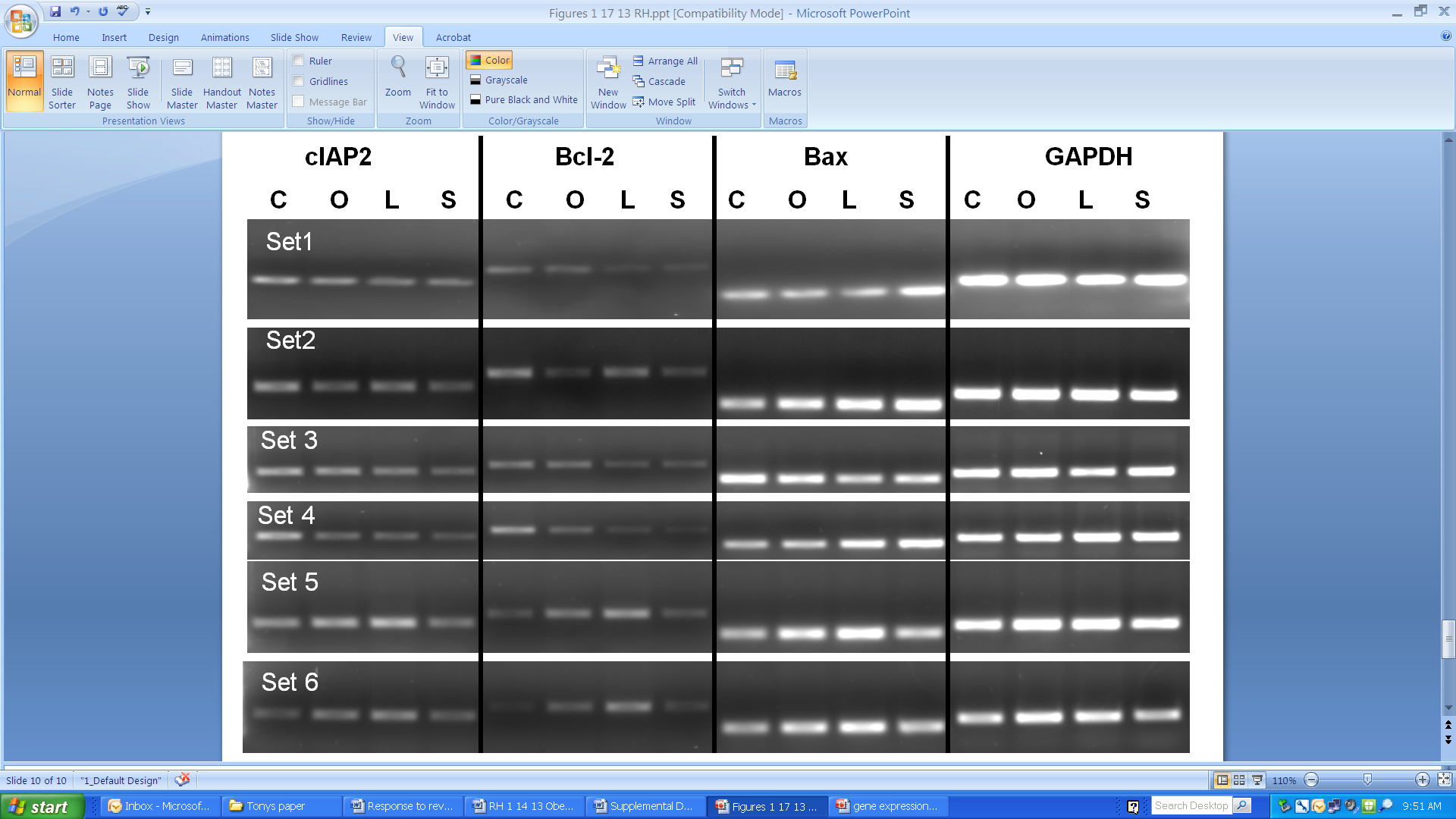


Figure S5. Effects of 50 µM stearic acid, oleic acid or linoleic acid on gene expression in 3T3L1 preadipocytes; C, control; O, oleic acid; L, linoleic acid; S, stearic acid. Control cells were treated identical to fatty acid treated cells except they were treated with fatty acid free BSA (see methods under Fatty Acids). Cells were treated for 48 hours. These data showed that stearic acid decreased the expression of cIAP2 and Bcl2 (although only cIAP2 was significant (n=6 replicates, p<0.01), which encodes antiapoptotic proteins, and increased Bax gene expression, which encodes for a proapoptotic protein, when compared to control cells. Linoleic acid also increased expression of Bax compared to control cells (n=6 replicates, p<0.01). These data are consistent with our apoptosis flow cytometry data and raise the possibility of a cIAP2 mediated mechanism of action for stearic acid induced apoptosis of preadipocytes.
